# Potency determination of inactivated H7 influenza vaccines using monoclonal antibody‐based ELISA and biolayer interferometry assays

**DOI:** 10.1111/irv.12528

**Published:** 2017-12-15

**Authors:** Anupama Vasudevan, Amy Woerner, Falko Schmeisser, Swati Verma, Ollie Williams, Jerry P. Weir

**Affiliations:** ^1^ Division of Viral Products Center for Biologics Evaluation and Research Food and Drug Administration Silver Spring MD USA

**Keywords:** A(H7N9), influenza, potency assay

## Abstract

**Background:**

The single radial immunodiffusion (SRID) assay, the accepted method for determining potency of inactivated influenza vaccines, measures an immunogenic form of the influenza hemagglutinin. Nevertheless, alternative methods for measuring vaccine potency have been explored to address some of the weaknesses of the SRID assay, including limited sensitivity and the requirement for large amounts of standardized reagents. Monoclonal antibody (mAb)‐based potency assays also have the ability to detect and measure relevant immunogenic forms of HA.

**Objectives:**

The objective of this study was to continue evaluation of mAb‐based alternative methods for measuring the potency of inactivated influenza vaccines, focusing on A(H7N9) pandemic influenza vaccines.

**Methods:**

Several murine mAbs that recognize different epitopes on the H7 hemagglutinin (HA) were identified and characterized. These mAbs were evaluated in both a mAb‐capture ELISA and a mAb‐based biolayer interferometry (BLI) assay.

**Results:**

Results indicated that potency of inactivated A(H7N9) vaccines, including vaccine samples that were stressed by heat treatment, measured by either alternative method correlated well with potency determined by the traditional SRID potency assay.

**Conclusions:**

The availability of multiple H7 mAbs, directed to different HA epitopes, provides needed redundancy in the potency analysis as A(H7N9) viruses continue to evolve antigenically and suggests the importance of having a broad, well‐characterized panel of mAbs available for development of vaccines against influenza strains with pandemic potential. In addition, the results highlight the potential of mAb‐based platform such as ELISA and BLI for development as alternative methods for determining the potency of inactivated influenza vaccines.

## 1 INTRODUCTION

Traditionally, the potency of inactivated influenza vaccines has been determined using the single radial immunodiffusion (SRID) assay, an agarose gel‐based format that uses strain‐specific polyclonal antibody reagents to quantify the amount of influenza hemagglutinin (HA) present in a vaccine sample by comparison with the assigned HA value of a reference antigen standard.^1^, ^2^, ^3^ The SRID assay is relatively simple and practical, strain‐specific, and has acceptable accuracy and robustness for current vaccines. However, the dynamic range of the SRID is limited, the assay may not be ideal for newer types of influenza vaccines, and the assay requires large amounts of calibrated reagents that must be produced in a timely manner to support vaccine manufacturing. The latter issue is always a concern, both in the context of seasonal influenza vaccine manufacturing, as well as in the response to the emergence of a pandemic influenza strain.^4^ Indeed, difficulties were encountered in the preparation of the SRID potency antisera for the A(H1N1)pdm09 pandemic vaccine and for candidate vaccines made in response to the emergence of the A(H7N9) virus in China in 2013.^5^

In an attempt to address some of the limitations of the SRID, several newer methods have been explored in recent years as possible alternative potency assays for inactivated influenza vaccines.^6^, ^7^, ^8^, ^9^, ^10^, ^11^, ^12^, ^13^ Several of these assays rely on the use of strain‐specific monoclonal antibodies (mAbs) to capture and quantify HA in vaccine samples. Although the initial reports describing mAb‐based alternative potency assays are promising and demonstrate the general feasibility for further development, there are unanswered questions concerning the identification and selection of the appropriate antibodies and how such antibody reagents can be generated in the time frame required for vaccine manufacture. These issues are especially concerning in the time frame of pandemic influenza vaccine manufacturing.

The goal of this study was to continue evaluation of mAb‐based alternative methods for measuring the potency of inactivated influenza vaccines, focusing on A(H7N9) pandemic influenza vaccines produced following the emergence of novel A(H7N9) viruses in China in 2013 that resulted in hundreds of human fatalities.^14^, ^15^ Several mAbs, recognizing different epitopes on the H7 HA, were identified, characterized, and evaluated in both a mAb‐capture ELISA and a mAb‐based biolayer interferometry (BLI) assay. The results indicated that potency of inactivated A(H7N9) vaccines, including vaccine samples that were stressed by heat treatment, measured by either alternative method correlated well with potency determined by the traditional SRID potency assay and suggested the value and feasibility of having a broad, well‐characterized panel of mAbs available for development of vaccines against influenza strains with pandemic potential. Overall, the results indicate the potential of mAb‐based ELISA and BLI platforms for continued development as alternative methods for determining the potency of inactivated influenza vaccines.

## 2 MATERIALS AND METHODS

### 2.1 Cells and viruses

The A(H7N9) A/Shanghai/2/2013 virus used in these studies is a reassortant candidate vaccine virus (RG32A) prepared by and obtained from the Centers for Disease Control and Prevention (Atlanta, GA, USA). Influenza viruses were propagated in 9‐day‐old specific pathogen‐free embryonated chicken eggs. Selection and characterization of A(H7N9) escape viruses were performed in Madin‐Darby canine kidney (MDCK) cells. Mammalian virus‐like particles (VLPs) containing the HA of the A(H7N9) A/Shanghai/2/2013 virus were prepared by modified vaccinia virus Ankara (MVA) vector infection of Vero cells and purified as previously described.^16^ All virus and VLP work was approved by the FDA's Institutional Biosafety Committee. Reference antigens for the A(H7N9) influenza vaccine virus were produced by the Center for Biologics Evaluation and Research (CBER)/FDA. All cells were maintained in Dulbecco's modified Eagle medium supplemented with 10% FBS (HyClone, Logan, UT, USA), 2 mM L‐glutamine, and 50 μg/mL gentamicin.

### 2.2 Production of influenza H7 monoclonal antibodies

Purified murine mAbs to A/Shanghai/2/2013 HA were prepared as previously described,^17^ using VLPs containing the HA from A/Shanghai/2/2013 as the immunogen. To select for mAbs directed to epitopes other than antigenic site A in HA, VLPs were prepared as immunogens from two modified MVA vectors that expressed either the H7 HA with a glycosylation site motif introduced at amino acid 123‐125 (amino acid numbering throughout the text refers to the mature H7 HA, excluding the HA N‐terminus signal peptide), or an H7 antigenic site A mutation at amino acid position 131 (R131G). Targeted mutations were introduced into the MVA plasmid insertion vectors using QuickChange Lightning^®^ Site‐Directed Mutagenesis Kit (Agilent Technologies, Santa Clara, CA, USA). Hybridoma clones secreting mAbs to influenza H7 HA were screened by ELISA using inactivated A/Shanghai2/2013 reference antigen as a capture antigen.

### 2.3 Selection of escape mutants

The selection of A(H7N9) escape virus mutants^18^ was performed by incubating A/Shanghai/2/2013 virus with H7 mAbs over a range of concentrations from 40 to 0.156 μg/mL, selecting resistant virus, and repeating the process for up to 2 more rounds of selection. Escape mutants were sequenced and tested for reduced inhibition of neutralization by the mAb compared to the parent virus.

### 2.4 Measurement of potency by mAb ELISA

Potency ELISAs were performed as previously described.^12^ Purified capture mAbs were used at a concentration of 2‐4 μg/mL (determined empirically for each mAb to optimize antigen capture and minimize non‐specific background). Reference antigen and vaccine samples were treated with 1% Zwittergent 3‐14 for 30 minutes, diluted in PBS/Tween/10% FBS (minimum 10‐fold additional dilution) before being added to the Immulon 2HB plate. The primary detection antibody was a purified rabbit polyclonal IgG, generated by the immunization of rabbits with plasmid DNA vectors expressing A/Shanghai/2/2013 HA and boosted with mammalian‐derived VLPs containing the same H7 HA. The secondary detection antibody was a goat anti‐rabbit IgG conjugated with HRP. A 1:1 mix of ABTS:H_2_O_2_ was used as enzyme substrate. The HA concentration was determined by parallel line analysis of the four‐parameter regression fits of vaccine samples to that of the standard (the reference antigen) on each plate. Replicates were included on each plate, and assays were repeated on different days.

### 2.5 Measurement of potency by single radial immunodiffusion

The SRID assay was performed as previously described.^19^, ^20^ Vaccine potency was calculated using the parallel line bioassay method, which uses reference and test vaccine dose‐response curves (log antigen dilution versus log zone diameter). Replicates were included in each SRID assay, and assays were repeated on different days.

### 2.6 Biolayer interferometry

Epitope binning and vaccine potency determination by BLI were performed on an Octet Red‐384 system (Pall ForteBio, Menlo Park, CA USA). For epitope binning, recombinant H7 (rHA) A/Anhui/01/2013 (Protein Sciences, Meriden, CT, USA) in PBS was biotinylated using an EZ‐Link NHS‐PEG_4_ Biotinylation kit (Thermo Fisher, Rockford, IL, USA). A/Anhui/01/2013 is an A/Shanghai/2/2013‐like A(H7N9) virus with the same HA as A/Shanghai/2/2013.

Binning was performed in 96‐well microplates (Pall ForteBio) and used High Precision Streptavidin‐coated (SAX) biosensors (Pall ForteBio) loaded with the biotinylated A/Anhui rHA at 5 μg/mL (determined empirically to generate a response signal of ~0.5). H7 antibodies were loaded into adjacent wells at a concentration of 50 μg/mL for initial saturating binding to HA and also loaded into a second set of wells at a concentration of 25 μg/mL to be used as the competing Ab in the assay. Loading time for the biotinylated rHA onto the SAX biosensors was 600 seconds; loading times for antibody 1 (saturating Ab) and antibody 2 (competing Ab) were 300 seconds. The binning experiments were designed so that every antibody was used for saturation and competition against all of the other antibodies. Data Analysis HT 9.0 software (Pall ForteBio) was used to analyze the results, which were presented in a matrix format to indicate antibody combinations that were either blocking or non‐blocking.

Biolayer interferometry vaccine potency determination experiments used Dip and Read Anti‐Mouse IgG Fc Capture (AMC) biosensors (Pall ForteBio) in a 384‐well plate format (tilted‐bottom microplates, Pall ForteBio) with a baseline buffer consisting of Kinetics Buffer (Pall ForteBio) with 0.1% Tween 20/0.1% BSA. Each mAb concentration was optimized by initially diluting the mAb to 10 μg/mL followed by twofold serial dilution before loading onto the AMC biosensor. Reference antigen, diluted to 30 μg/mL, was bound to the different concentrations of mAb, and the optimal mAb binding concentration was determined by selecting the highest mAb concentration at which the binding curves were not overlapping. For mAbs 1E9, 7B5, and 98, the optimal mAb concentration was determined to be 0.3 μg/mL. For mAb 5A6, an optimal concentration of 0.128 μg/mL was determined.

For vaccine potency determination, the AMC biosensors were dipped into buffer (baseline step) for 60 seconds, followed by loading the optimized mAb concentration for 300 seconds (load step). The biosensors were then dipped into the baseline buffer again, followed by the reference standard for 300 seconds (association step). A separate set of biosensors was used to repeat this process, except that the association steps used vaccine samples. The reference antigen and vaccine samples were prepared as a twofold dilution series with a starting concentration of approximately 30 μg/mL. All steps were performed at 23°C at a shake speed of 400 rpm. The HA concentration of vaccine samples was calculated by comparing the standard curve of the reference antigen to the standard curve generated for each vaccine sample. The same read time was used for both the reference and the vaccine sample being compared (20‐300 seconds), and an unweighted dose‐response 4PL curve was used for both the reference and the vaccine samples. Three replicates of standards and unknowns were included on each plate, and each assay repeated a minimum of two times on different days.

## 3 RESULTS

### 3.1 Isolation and characterization of monoclonal antibodies to the influenza H7 hemagglutinin

In a previous study, we described the isolation and characterization of murine mAbs to the H7 HA of the recently emerged A(H7N9) viruses in China,^17^ but interestingly, all of the mAbs isolated in that study were directed to antigenic site A. In order to broaden the H7 HA epitope representation of our mAbs, we generated and characterized additional panels of mAbs using approaches designed to select for mAbs directed to epitopes other than antigenic site A. In addition, we evaluated some existing mAbs (mAbs 62 and 98) developed to an older H7N1 strain to determine how well these mAbs would bind the more recent A(H7N9) hemagglutinins.^21^, ^22^ Monoclonal antibodies were assessed for binding to HA in an ELISA using inactivated A(H7N9) A/Shanghai/2/2013 virus. Several mAbs that bound H7 HA well were identified and selected for further characterization, including testing for hemagglutination inhibition and binding in Western blot under reducing and non‐reducing conditions (Table 1).

**Table 1:** Characterization of H7 monoclonal antibodies

Antibody	Binding titer by ELISA^a^	Hemagglutination inhibition titer^b^	Western blotreducing/non‐reducing^c^
1A10	12 821K	3238	+/+
1E9	3226K	481	‐/+
5A6	1587K	241	‐/+
7B5	6250K	3851	‐/+
7E3	3226K	1925	+/+
62	1587K	6476	‐/+
98	1587K	4579	‐/+

a Endpoint titer—highest dilution of antibody (initial concentration of 4 mg/mL) giving an absorbance value (405 nm) >0.050 and greater than the highest dilution of a matched dilution of control antibody of the same isotype; K = 1000; antigens for capture (inactivated whole influenza A/Shanghai/2/2013 virus used at 10 μg/mL).

b GMT of the antibody inhibition of A/Shanghai/2/2013 hemagglutination of chicken red blood cells; initial mAb concentration 0.8 mg⁄ mL.

c mAb binding of A/Shanghai/2/2013 HA in Western blot analysis under reducing and non‐reducing conditions.

### 3.2 Epitope analysis of influenza A(H7N9) monoclonal antibodies

Biolayer interferometry was used to perform epitope binning of the H7 A/Shanghai mAbs using biotinylated H7 A/Anhui/1/2013 rHA. Each mAb was captured separately to saturation, and all other mAbs were used as competing antibodies in order to examine competitive binding. As shown in Table 2, three of the mAbs (7B5, 7E3, and 1A10) grouped in one antigenic “bin” that did not include the other four mAbs (1E9, 98, 5A6, and 62). Of these other four mAbs, 1E9, 98, and 5A6 clearly grouped into a second antigenic “bin”, as using any of these mAbs as saturating antibody blocked binding of itself and the other mAbs. The results for mAb 62 were not as clear‐cut, but suggested that mAb 62 is also likely part of the second “bin.” Although mAb 62 competed somewhat with saturating 1E9, 98, and 5A6 mAbs, saturating mAb 62 completely blocked subsequent binding by itself as well as 1E9, 98, and 5A6 (column 62).

**Table 2:** Epitope binning of H7 monoclonal antibodies by biolayer interferometry

mAb^a^	Saturating mAb^b^							
	7B5	7E3	1A10	1E9	98	5A6	62	H5 2C6
7B5	0.0103	0.0133	0.0081	0.3313	0.3983	0.2563	0.1542	−0.0378
7E3	0.0097	0.0108	0.0085	0.3177	0.3918	0.2586	0.1542	−0.0368
1A10	−0.0022	−0.0013	0.0091	0.3324	0.404	0.2774	0.1763	−0.0271
1E9	0.2266	0.2311	0.2137	0.0176	0.0194	−0.0235	−0.0752	−0.1449
98	0.2775	0.278	0.2835	0.0551	0.0345	0.0211	−0.0629	−0.095
5A6	0.2009	0.1989	0.1799	0.0605	0.0686	0.014	−0.0752	−0.1967
62	0.288	0.2918	0.3048	0.1954	0.1578	0.1404	0.0142	−0.0544
H5 2C6	0.4263	0.4378	0.3934	0.3993	0.4355	0.3149	0.2337	−0.0039

a Competing mAb—Data presented is the raw nanometer shift caused by the binding of the competing antibody. To differentiate between competing antibodies that are blocked by the saturating antibody and those that are not, a threshold equal to the highest self‐binding signal in the panel is set: 0.0345 (mAb 98). The threshold value is then used to color‐code the matrix data in either red or green, to distinguish between competing antibodies that are or are not blocked by the saturating antibody.

b Saturating mAb—H7 antigen was captured onto the SAX sensor surface using biotin tag and the loaded sensor first exposed to the indicated saturating mAb.

**Figure 1:**
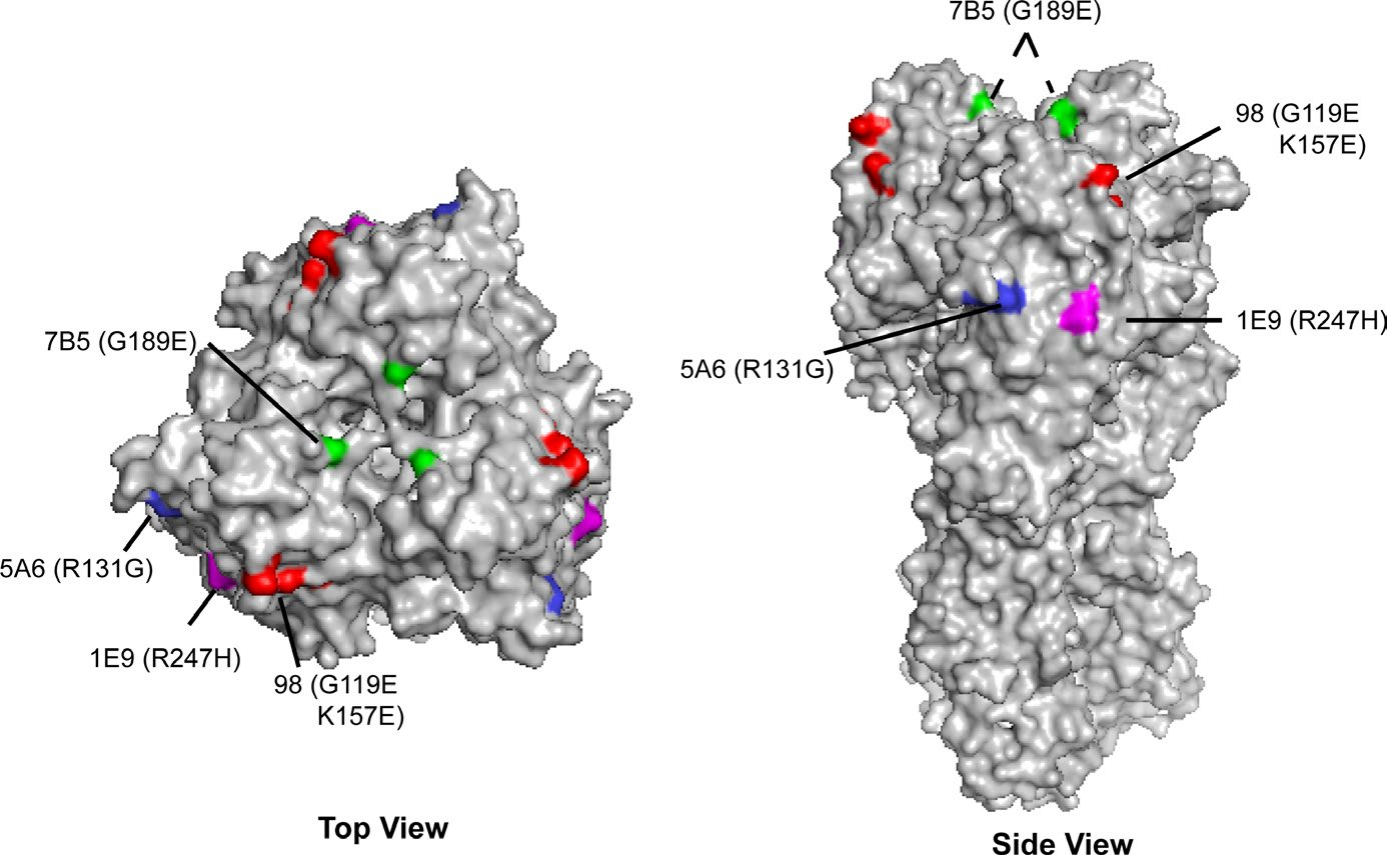
Location of HA amino acid changes in influenza A(H7N9) escape mutants. Antigenic structure of the A(H7N9) A/Shanghai/2/2013 HA trimer (PDB ID: 4LN6) and location of the escape mutations to mAbs 7B5 (green), 5A6 (blue), 1E9 (magenta), and 98 (red). A, Top view—the location of each escape mutation on one HA molecule of the trimer is indicated. B, Side view—the location of three escape mutations (5A6, 98, and 1E9) are shown on one HA monomer of the HA trimer; the location of the 7B5 mutation on the other two HA monomers is shown by dotted lines

Finer mapping of the epitopes recognized by the H7 mAbs was performed by generating virus escape mutants. Escape mutant viruses with single amino acid changes were obtained for mAbs 7B5 and 1A10 (G189E) and 1E9 (R247H). The G189 mutation is near antigenic site B, defined originally for influenza H3 HA;^23^, ^24^ the mutation at position 247 selected by mAb 1E9 is not in any of the previously defined antigenic sites for influenza H3. The locations of these escape mutations, as well as those for 5A6^17^ (previously mapped to antigenic site A – R131G), are shown in Figure 1. Escape mutant viruses with amino acid changes at positions 119 (G119E) and 157 (K157E) have previously been reported for mAbs 98^22^ and 62,^21^ respectively. In several attempts at isolating escape mutants to these two mAbs, viruses with both amino acid changes (G119E and K157E) were always obtained. As shown in Figure 1, these two amino acids are located spatially adjacent to each other on the HA, suggesting that mAbs 62 and 98 probably recognize the same HA epitope.

Cross‐neutralization experiments with the escape mutant viruses and the H7 mAbs were used to extend the epitope analysis of the H7 mAbs (Table 3). Initial experiments confirmed that mAbs 7B5, 1A10, and 7E3 shared a common epitope, as none of these mAbs were able to neutralize an escape virus with a G189E mutation (data not shown). In contrast, mAbs 5A6, 1E9, and 98 easily neutralized the G189E escape virus and mAb 7B5 easily neutralized the other three escape viruses. Although 5A6 and 1E9 grouped together in the BLI binning experiments, neutralization analysis indicated that the mAbs were different, as 5A6 was capable of neutralizing the 1E9 escape virus and 1E9 was capable of neutralizing the 5A6 escape virus. All escape viruses were neutralized well by mAb 98 indicating that the epitope recognized by this mAb differed from the other mAbs, including mAbs 5A6 and 1E9 which were grouped together with mAb 98 by BLI binning. The mAb 98 escape virus was not neutralized by mAb 1E9, however, suggesting that the 1E9 and 98 epitopes, although different, might be spatially close to each other on HA. Taken together, four distinct epitopes on the H7 HA were identified by the mAbs characterized in this study. Three epitopes, recognized by mAbs 5A6, 1E9, and 98, are located on the outer face of each HA monomer, whereas the fourth epitope recognized by mAb 7B5 is closer to the receptor‐binding site of HA (Figure 1).

**Table 3:** mAb neutralization of A/Shanghai/2/2013 escape mutants

Virus^a^	mAb^b^			
	5A6	7B5	1E9	98
A/Shanghai/2/2013	+++	+++	+++	+++
5A6v (R131G)	‐	++	++	+++
7B5v (G189E)	++	‐	++	+++
1E9v (R247H)	+	+++	‐	+++
98v (G119E/K157E)	+	++	‐	‐

a Each virus was titrated and diluted to approximately 500 pfu/mL and incubated with mAb concentrations from 80 to 0.31 μg/mL for incubation with mAb.

b (‐) No virus neutralization at mAb >80 μg/mL; (+) neutralization at 20 μg/mL; (++) neutralization at 5 μg/mL; (+++) neutralization at mAb between 0.31 and 1.25 μg/mL.

### 3.3 Potency determination of A(H7N9) inactivated influenza vaccines by mAb‐capture ELISA and biolayer interferometry

Monoclonal antibodies to H7 HA were used to develop a capture ELISA for quantifying the HA content of inactivated influenza A(H7N9) vaccines. The assay setup was similar to that previously described^12^ and used four different H7‐specific capture mAbs. ELISA potency values were determined by comparing the binding of HA in the vaccine samples relative to the binding of the reference antigen standard that has an assigned value in μg of HA. The measured ELISA potency values were compared to potency values determined concurrently by SRID assay. Two inactivated H7 vaccine samples from 2 different vaccine manufacturers were available for evaluation by SRID and mAb‐capture ELISA.

**Figure 2:**
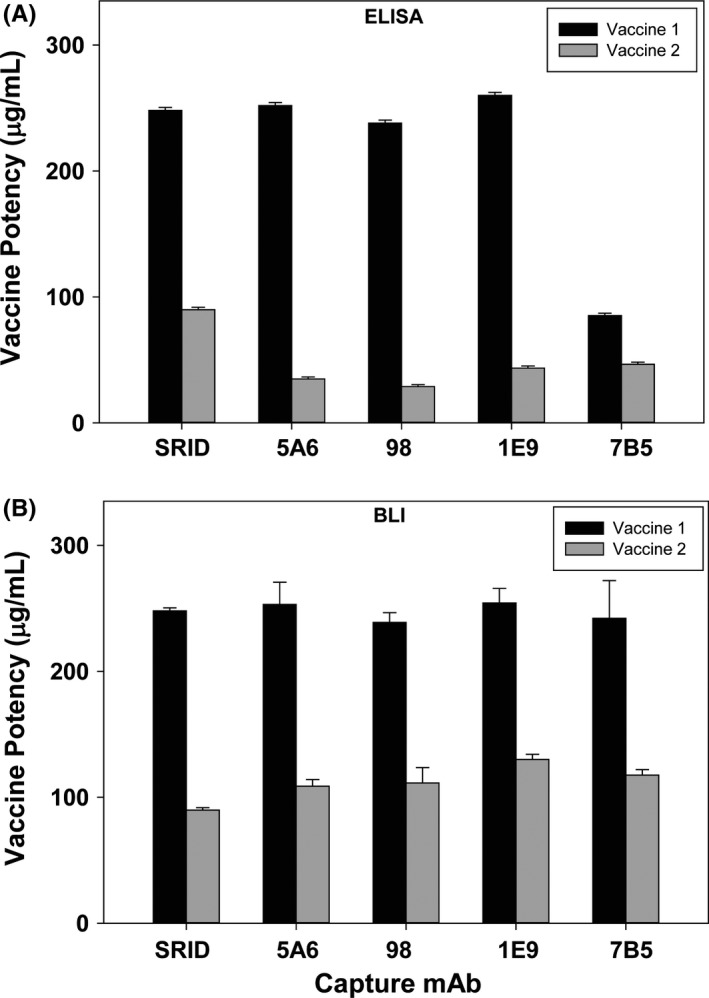
Potency values of two inactivated A(H7N9) vaccines determined by SRID, ELISA, and BLI. A, Potency and standard deviation of A/Shanghai/2/2013 A(H7N9) vaccines from two manufacturers were determined by traditional SRID analysis and ELISA using four H7‐specific mAbs. B, Potency and standard deviation of A/Shanghai/2/2013 A(H7N9) vaccines from two manufacturers were determined by traditional SRID analysis and BLI using four H7‐specific mAbs

The potency values obtained for Vaccine 1 using the ELISA‐based potency assay were similar for mAbs 5A6, 98, and 1E9 (Figure 2A), ranging from 238 to 260 μg/mL, and similar to the SRID value of 248 μg/mL. However, the potency value obtained using mAb 7B5 was approximately 66% lower (85.1 μg/mL) than the average of the other three mAbs (250 μg/mL), suggesting that mAb 7B5 interacted differently with Vaccine 1 relative to binding of the reference antigen in the ELISA format. For Vaccine 2, the potency values determined using all four mAbs were similar, averaging 38 μg/mL (Figure 2A), but was approximately 57% lower than the corresponding SRID potency value of 90 μg/mL, suggesting that the mAbs did not capture this vaccine as well as the reference antigen in the ELISA format.

In the mAb‐capture ELISA potency assay, HA is quantified by comparing the total amount of HA in a vaccine and a reference standard bound by the capture mAbs. However, the rate of binding to the antibody should also be dependent on the concentration of HA in the sample and can be measured by techniques such as biolayer interferometry. We explored the development of a BLI assay as another alternative assay to quantify HA in vaccine samples in comparison with a reference standard using the four H7‐specific mAbs described above. Preliminary experiments determined a loading concentration for each mAb and generated response curves to verify that the binding rate of reference antigen and vaccine samples to the mAb on the biosensor is concentration dependent (Materials and Methods).

Biolayer interferometry potency values were determined by generation of a binding rate response curve using dilutions of the reference antigen (with an assigned value of HA in μg) to each H7 mAb and comparing that standard curve to a similar binding rate response curve for each of the two inactivated vaccine samples. The potency values obtained for Vaccine 1 using the BLI‐based potency assay were consistent for the four mAbs, ranging from 238 μg/mL (mAb 98) to 254 μg/mL (mAb 1E9), and also correlated well with SRID value of 248 μg/mL for this vaccine sample (Figure 2B). Similarly, potency values obtained for Vaccine 2 were consistent for the four mAbs used in the assay, ranging from 109 μg/mL (mAb 5A6) to 130 μg/mL (mAb 1E9), and correlated well with the SRID value for Vaccine 2 of 90 μg/mL.

### 3.4 Potency of temperature‐stressed A(H7N9) A/Shanghai vaccine

To determine whether the mAb‐based ELISA and BLI assays were able to distinguish subpotent A(H7N9) vaccine and accurately quantify loss of potency, we employed heat treatment at 56°C to accelerate the decline in potency and establish conditions under which potency was significantly reduced. Aliquots of A(H7N9) Vaccine 1 were incubated at 56°C for 15 minutes, 1, 4, and 24 hours and then were assayed by SRID, as well as ELISA and BLI using 1E9, 7B5, 5A6, and 98 H7 mAbs (Table 4). As measured by SRID, this vaccine exhibited a rapid loss of potency over time, with an 82% decline in potency after only 15 minutes at 56°C. The potency continued to decrease over time until the HA content was undetectable at 24 hours.

**Table 4:** Potency of A(H7N9) vaccine subjected to temperature stress at 56°C determined by SRID, ELISA, and BLI

Time at 56°C (h)	SRID	ELISA % Unstressed potency a					BLI % Unstressed potency				
		All mAbs^b^	mAb 1E9	mAb 7B5	mAb 5A6	mAb 98	All mAbs	mAb 1E9	mAb 7B5	mAb 5A6	mAb 98
0	100	100	100	100	100	100	100	100	100	100	100
0.25	18	26	19.7	44	22.7	19.2	3.4	3.4	5	2.4	2.6
1	10.9	3.2	2	5.6	3.1	2.1	1.2	1.2	1.3	1.0	1.2
4	5.6	0.9	0.92	1.4	0.4	0.76	N.D.	N.D.	N.D.	N.D.	N.D.
24	N.D.	N.D.	N.D.	N.D.	N.D.	N.D.	N.D.	N.D.	N.D.	N.D.	N.D.

a N.D.—Percent unstressed potency of vaccine relative to 4°C.

b N.D.—Average of all 4 mAb potency values.

N.D., Not detectable.

There was also a decline in potency as measured by ELISA and BLI using each H7 mAb. The relative potency decline measured by ELISA and BLI generally mirrored the decline in potency as measured by SRID, and the results obtained in both the ELISA and BLI analyses were similar for all four mAbs in each assay, indicating that all H7 mAbs were stability‐indicating in these assays. The potency decline measured by ELISA or BLI appeared somewhat more rapid than that measured by SRID, particularly for the BLI analysis. For example, there was no detectable HA by the 4‐hour time‐point in the BLI analysis using any mAb. Taken together, the data show that similar heat‐stressed declines in potency can be measured by all three assay platforms (SRID, ELISA, BLI) with all of the H7 mAbs and that all the platforms are capable of distinguishing temperature‐stressed vaccines from unstressed vaccines.

## 4 DISCUSSION

The SRID assay is the accepted standard for determining the potency of inactivated influenza vaccines. Importantly, the assay measures an immunogenic form of the HA antigen in the vaccine, and a link between SRID potency and vaccine immunogenicity and vaccine efficacy has been established. Nevertheless, the limitations of the SRID assay have spurred development of alternative methods to measure influenza vaccine potency. Monoclonal antibody‐based potency assays also have the ability to detect and measure relevant immunogenic forms of HA, and several such promising assays have been described. However, there are at least two key issues that will have to be resolved during the development of mAb‐based potency assays. One issue is the selection of the appropriate mAbs for the assay, including whether multiple antibodies are necessary to accurately assess the potency of the HA antigen in the vaccine. A second issue is whether, and how, suitable mAbs can be generated and characterized in the time frame of either seasonal or pandemic influenza vaccine manufacturing so that antibody production is not a potential bottleneck to timely vaccine manufacture.

In the current study, we began addressing these two issues in the context of mAb‐based potency assays for pandemic H7 influenza vaccines that were developed following the A(H7N9) outbreak in China in 2013. Since the initial outbreak, waves of A(H7N9) virus infections in humans have reappeared each winter season, resulting in numerous human infections and deaths.^25^ We generated and characterized several mAbs recognizing different epitopes on the H7 HA and evaluated these mAbs, as well as H7 mAbs (mAbs 62 and 98) that were developed several years prior to the A(H7N9) outbreak in China, for potency determination of A(H7N9) vaccines using two assay formats. Potency results from these assays were compared to the potency results obtained using the SRID potency assay. There was generally good correlation between the SRID potency values and potency values obtained using either the mAb‐capture ELISA or BLI assay for most of the vaccine samples tested. However, as has been observed and discussed previously,^9^, ^26^ there are occasionally discrepancies in the actual values determined by SRID and any alternative potency assay, which may be at least partially related to the type of reference antigen used in the comparative analyses; additional work will be needed to better understand and resolve this issue. In the potency ELISA results reported here, one mAb (7B5) yielded potency results that were significantly lower for Vaccine 1 than the other tested mAbs. This difference in potency was not observed in the ELISA with the other vaccine sample, nor in the BLI assay with either Vaccine 1 or Vaccine 2, indicating that there are differences in the way individual mAbs interact with HA antigen in different vaccine formulations or assay formats. Although the fact that a particular mAb behaves differently in two assay formats may not be especially surprising, it does emphasize the importance of the issue of mAb redundancy and mAb selection and characterization, the criteria for which at this point in time remain mostly empirical. Importantly, however, mAbs such as mAb 98 that were generated to an earlier H7N1 strain, before the emergence of the A(H7N9) viruses in 2013, worked well in both assay formats to quantify HA in the available A(H7N9) vaccine samples. In addition, recent studies have indicated that some of the mAbs in the current panel of H7 mAbs, including 7B5 and 1E9, will capture HA from recent strains of A(H7N9) (e.g, A/A/Hong Kong/125/2017 and A/Guangdong/17SF003/2016) that are being developed as newer candidate vaccines (data not shown). Taken together, these results suggest the importance of evaluating multiple capture mAbs, including those directed to different HA epitopes, early in assay setup and development to increase the robustness of the assay, and the necessity of developing panels of mAbs to HAs of potential pandemic influenza viruses, as well as for seasonal influenza strains, as a practical solution for implementation of a timely potency assay using mAbs.

Previous studies have shown that the ELISA‐based mAb approach to determining the potency of vaccines can be used for a variety of vaccines and strains, although only a limited amount of development work has been performed for candidate pandemic vaccines. The ELISA approach as an alternative influenza vaccine potency assay has several attractive advantages. It is a relatively simple method commonly used in laboratories worldwide, requires greatly reduced amounts of standardized reagents, and is amenable to high‐throughput automation. In addition, the ELISA method is more sensitive than the SRID and has a shorter assay time. The ELISA potency assay does require strain‐specific antibodies, however, as well as characterized detection antibodies, and as shown in the current study and previous studies, the selection of the most appropriate antibodies for the assay is still empiric, and developmental work is necessary to set up the assay for a particular strain and vaccine formulation. For example, in addition to the poor capture of Vaccine 1 by mAb 7B5 in the ELISA format, other H7‐specific mAbs such as 1A10 and 7E3 (Table 1) that recognize the same HA epitope as 7B5 did not capture H7 reference antigen very well in the ELISA setup (data not shown).

More recently, biolayer interferometry has emerged as an assay platform for protein quantification and its potential for determination of the potency of inactivated influenza vaccines has been proposed.^27^ We used BLI to measure HA by comparing the binding of HA in vaccine samples to specific mAbs in comparison with a reference standard. In contrast to the ELISA, however, BLI measures rates of binding rather than total HA binding and does not require a detection antibody step. This method is of high throughput and is extremely fast, allowing for multiple samples and replicate assays to be run each day. Furthermore, fewer steps are required for the BLI assay compared to the ELISA method because no additional detection steps or reagents are required, although without an additional amplification step, the BLI method is less sensitive than the ELISA. We set up the BLI assay using anti‐mouse IgG Fc biosensors to bind the H7 mAbs, but other biosensor presentations for the mAbs, as well as amplification steps, are possible. Although there is far less collective experience with a BLI‐based assay than ELISA‐based assays for vaccine potency determination, the initial studies are encouraging and the rapid turnaround time and high‐throughput capability are particularly appealing. Further studies will be needed to determine whether this type of assay can be developed as an alternative potency assay for influenza vaccines.

Encouragingly, both alternative potency assay formats using H7‐specific mAbs were capable of distinguishing heat‐stressed vaccine samples from non‐stressed samples. Both alternative assays measure fairly rapid declines in potency when vaccine was subjected to heat treatment at 56°C and were, in fact, somewhat more sensitive to the heat treatment than the SRID assay (e.g, 1‐ and 4‐hour time‐points). Future studies will be needed to further define the most appropriate methods for assessing the stability‐indicating capabilities of alternative assays, and this will be an important component of the evaluation and selection of antibodies for any mAb‐based assay.

In summary, the results of the current study broaden our understanding of the issues that must be resolved as development of mAb‐based alternative potency assay for influenza vaccines progresses. Although the preparation and characterization of strain‐specific mAbs will be a challenge, the results from this and other recent studies demonstrate that development and selection of cross‐reactive mAbs is a realistic possibility. Advanced development and preparation of a well‐characterized, diverse panel of mAbs that recognize different HA epitopes for influenza subtypes with pandemic potential, such as the H7 mAbs described in the present study, greatly increase the probability of having mAbs available for vaccine testing as influenza strains evolve.
